# The fungus-growing termite *Macrotermes natalensis* harbors bacillaene-producing *Bacillus* sp. that inhibit potentially antagonistic fungi

**DOI:** 10.1038/srep03250

**Published:** 2013-11-19

**Authors:** Soohyun Um, Antoine Fraimout, Panagiotis Sapountzis, Dong-Chan Oh, Michael Poulsen

**Affiliations:** 1 Natural Products Research Institute, College of Pharmacy, Seoul National University, 1 Gwanak-ro, Gwanak-gu, Seoul 151-742, Republic of Korea; 2 Centre for Social Evolution, Section for Ecology and Evolution, Department of Biology, University of Copenhagen, Universitetsparken 15, DK-2100 Copenhagen Ø, Denmark; 3 Current address: Muséum National d'Histoire Naturelle, UMR CNRS 7205 OSEB, Département Systématique et Evolution, 45 rue Buffon, 75005 Paris, France

## Abstract

The ancient fungus-growing termite (Mactrotermitinae) symbiosis involves the obligate association between a lineage of higher termites and basidiomycete *Termitomyces* cultivar fungi. Our investigation of the fungus-growing termite *Macrotermes natalensis* shows that *Bacillus* strains from *M. natalensis* colonies produce a single major antibiotic, bacillaene A (1), which selectively inhibits known and putatively antagonistic fungi of *Termitomyces*. Comparative analyses of the genomes of symbiotic *Bacillus* strains revealed that they are phylogenetically closely related to *Bacillus subtilis*, their genomes have high homology with more than 90% of ORFs being 100% identical, and the sequence identities across the biosynthetic gene cluster for bacillaene are higher between termite-associated strains than to the cluster previously reported in *B. subtilis*. Our findings suggest that this lineage of antibiotic-producing *Bacillus* may be a defensive symbiont involved in the protection of the fungus-growing termite cultivar.

Beneficial symbiotic associations between prokaryotes and eukaryotes are widespread in nature^1^. Such mutualistic relationships include defensive symbioses, which often involve selective antibiotics produced by prokaryotes against host antagonists^2^,^3^. Recent studies exploring chemical mediators of symbiotic interactions in beetles^4^,^5^, fungus-growing ants^6^,^7^,^8^, marine sponges^9^, and solitary wasps^10^ have provided insights into the fundamental functions of natural antibiotics, as well as the discovery of novel bioactive small molecules genetically coded by insect-associated microorganisms^6^,^7^,^8^,^9^,^10^,^11^,^12^.

Fungus-growing termites (Blattodea, Macrotermitinae) are major decomposers in the Old World tropics, where they form some of the most complex colony and mound structures known (Fig. 1a). The success of the Macrotermitinae is undoubtedly attributed to their engagement in a mutualistic symbiosis with *Termitomyces* fungi (Basidiomycota: Agaricales: Lyophyllaceae), which aid in the degradation of plant material^13^,^14^,^15^. The fungus is housed on a special substrate (fungus comb) in the nest, which is maintained by the termites through the continuous addition of partially digested plant material that has passed through the termite gut along with asexual *Termitomyces* spores^16^,^17^ (Fig. 1b). In return for continuous provisioning of a substrate for growth, *Termitomyces* serves as a nitrogen-rich food source for the termites. The association originated more than 35 million years ago and none of the more than 350 species of fungus-growing termites, or the fungus symbionts they maintain, have abandoned this long-term association^18^,^19^,^20^.

The success of termite fungiculture is expected to rely on the termites effectively defending both themselves and their cultivar fungus from invading competitors and diseases. The maintenance of the cultivar fungus in monoculture within individual nests^21^,^22^,^23^ is predicted to make the fungus prone to exploitation in the absence of the termites^24^,^25^ (Fig. 1c), but only few candidate antagonists of the symbiosis have been identified. Ascomycete fungi in the subgenus *Pseudoxylaria*^26^,^27^,^28^ (Fig. 1d) are prevalent in fungus-growing termite nests^27^ and appear to compete with *Termitomyces* for the substrate provided by the termites^29^, and *Trichoderma* will rapidly overgrow the termite fungus when termite workers are absent^30^.

Whether or not *Pseudoxylaria* and *Trichoderma* act as specialized disease-causing microbes in the fungus-growing termite symbiosis is not clear; however, their competitive and/or antagonistic potential against *Termitomyces* supports that it would be beneficial for the termite-fungus association to assure that they are suppressed. Obligate gut passage of the substrate prior to incorporation in the fungus comb may aid this, because this mode of substrate incorporation may allow for the selective inhibition of antagonists before entry to the fungus comb^17^.

Previous work in *Odontotermes formosanus* fungus-growing termites has suggested that gut- and fungus comb-residing *Bacillus* sp. may aid in the suppression of antagonistic fungi (*Trichoderma*) present in the substrate provided by the termites for *Termitomyces* growth. The study found that *Trichoderma* rapidly overgrows *Termitomyces in vitro*, that *Bacillus* sp. present in the termite gut and fungus comb produce a secretion that *in vitro* inhibits *Trichoderma* but not *Termitomyces* growth, but the compound responsible for this selective inhibition has remained unsolved^30^. Through bioassay-guided chemical analyses of *Bacillus* strains isolated from different colonies of *Macrotermes natalensis*, we show that termite-associated *Bacillus* strains produce a single major compound, bacillaene (**1**), which selectively inhibits known (*Pseudoxylaria* and *Trichoderma*) and potentially competitive or antagonistic (*Coriolopsis*, *Umbelopsis* and *Fusarium*) fungi that had been obtained in culture from *M. natalensis* colonies and, hence, may represent potential competitors or antagonists of *Termitomyces*. Whole-genome analyses of these strains revealed that they are phylogenetically close to *Bacillus subtilis* and have nearly identical genomes, including across the entire ca. 80 kb gene cluster coding for the production of bacillaene.

## Results

### Isolation and antifungal testing of symbiotic *Bacillus* from termites

**Figure 1:**
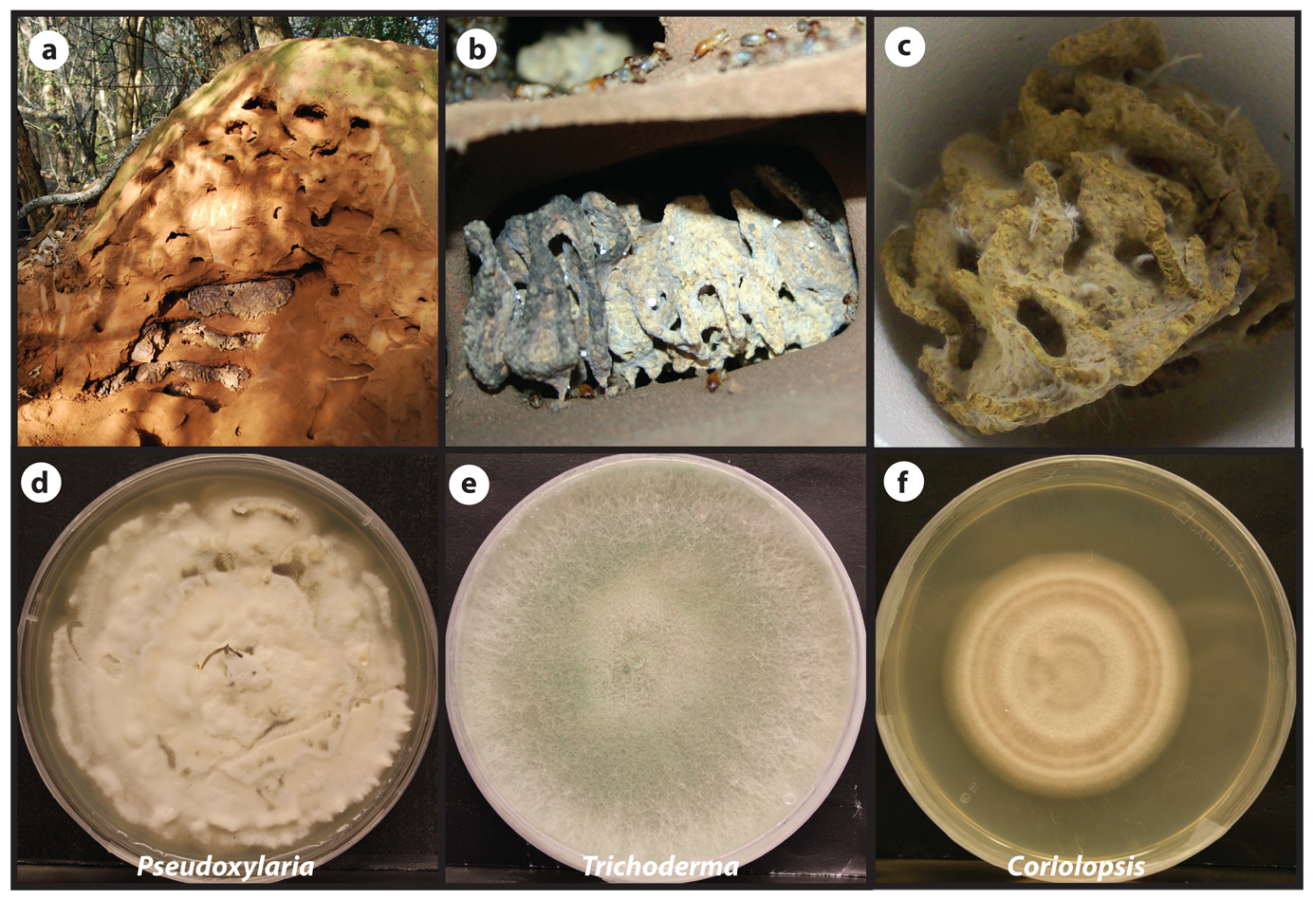
(a) *Macrotermes natalensis* fungus-growing termite colony. (b) A close up of the fungus comb (courtesy of Duur K. Aanen), generated by the termites through a mix of plant biomass and *Termitomyces* spores after termite gut passage, likely allowing for gut bacteria to control what enters the fungus comb. (c) In the absence of termites, the fungus comb is rapidly over-grown by *Pseudoxylaria*. (d–f) *In vitro* growth characteristics of three fungi isolated from *M. natalensis* fungus combs: (d) *Pseudoxylaria*, (e) *Trichoderma* and (f) *Coriolopsis* ((e) and (f), courtesy of Saria Otani).

Three *Bacillus* sp. strains (hereafter designated #9, #11 and #13) were isolated each from a different *Macrotermes natalensis* colony in Mookgophong, South Africa (S24°40′30.5″E28°47′50.4″). Isolates were obtained by the application of termite colony material (#9: fungus comb, #11: worker abdomen crushed in water, and #13: worker washed in water) on low-nutrient medium using standard isolation techniques. To explore the presence of antifungal activity, testing of the *Bacillus* strains was performed against *Termitomyces*, *Pseudoxylaria* (Fig. 1d) as well as four additional fungi isolated from the same termite species in addition to the beneficial *Termitomyces* fungus: *Trichoderma* sp. (Fig. 1e), *Coriolopsis* sp. (Fig. 1f), *Umbelopsis* sp. and *Fusarium* sp. Each bacterial strain was cultivated with each of the fungi on YEME agar plates. Seven days after inoculation, all three *Bacillus* strains inhibited the growth of all fungi except *Termitomyces*, suggesting the presence of a selective antifungal compound, which prompted us to make further efforts to identify the responsible compound.

### Chemical identification of the antifungal compound

To find the compounds from the *Bacillus* strains responsible for the growth inhibition of the five fungi, we cultivated *Bacillus* strains in YEME liquid culture medium. An initial LC/MS (liquid chromatography and mass spectrometry) analysis of the ethyl acetate (EtOAc) extract of the cultures revealed a common major secondary metabolite in all three strains, and this compound displayed the typical polyene UV spectral feature (λ_max_ at 346, 364, and 384 nm) and the low-resolution molecular ion [M + H]^+^ at *m*/*z* 581. Scaling up the culture conditions to 24 L of each of the *Bacillus* strains allowed for bioassay-guided fractionation to narrow down the active antifungal component in the extracts. The dried extract was fractionated under step gradient conditions using aqueous methanol (20, 40, 60, 80 and 100%) by open column reversed-phase chromatography on C_18_ resin. Each fraction was tested against *Pseudoxylaria*, *Trichoderma*, *Coriolopsis*, *Umbelopsis* and *Fusarium* using paper disk diffusion assays to trace active fractions.

**Figure 2:**
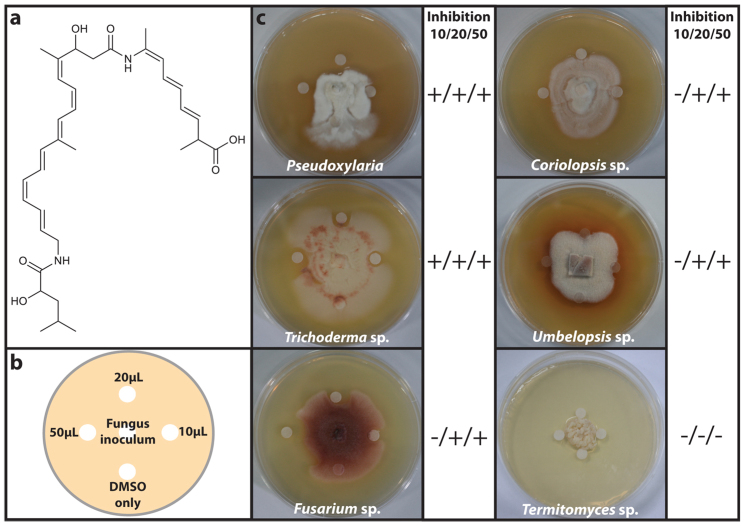
(a) The chemical structure of bacillaene A (**1**). (b) Petri plate antifungal activity assay setup, showing the placement of three concentrations (10 μL, 20 μL, 50 μL) of **1** (10 mg/mL) dissolved in DMSO, the control (DMSO only, 10 μL) and the placement of the fungal inoculum. (c) Representative image examples of bacillaene A activity against *Pseudoxylaria* 10 days after inoculation, *Trichoderma* sp. 3 days after inoculation, *Fusarium* sp. 3 days after inoculation, *Coriolopsis* sp. 5 days after inoculation, *Umbelopsis* sp. 7 days after inoculation, and *Termitomyces* 20 days after inoculation in addition to qualitative indications of the presence/absence (+/−) of inhibition at concentrations 10 μL, 20 μL, and 50 μL of **1** (10 mg/mL) for the five contaminant fungi as well as *Termitomyces*.

The antifungal assays demonstrated that the 80% aqueous MeOH fraction of each *Bacillus* culture was the most active, and LC/MS analysis of the fraction revealed the common major compound initially detected in the crude extract. Purification of the compound by preparative reversed-phase high performance liquid chromatography (HPLC) yielded the pure compound (**1**), which possessed the molecular formula C_34_H_48_N_2_O_6_ based on electrospray high-resolution mass spectrum ([M + H]^+^ at *m*/*z* 581.3585). Subsequently, we analyzed ^1^H and two-dimensional NMR spectra of compound **1**, specifically ^1^H-^1^H correlation (COSY; Fig. S3), heteronuclear single quantum coherence (HSQC; Fig. S4), and heteronuclear multiple bond correlation (HMBC; Fig. S5). The spectroscopic analysis and literature search identified compound **1** as bacillaene A, a polyene polyketide secondary metabolite^31^ (Fig. 2a).

We confirmed that bacillaene A is responsible for the antifungal activity observed using an antifungal assay (Fig. 2b). Petri dishes were observed daily for 30 days (Fig. 2c; Fig. S1). Bacillaene A (**1**) inhibited the growth of the fungi in a dose-dependent manner (Fig. 2c).

### Genomic identification of the bacillaene biosynthetic gene cluster

**Figure 3:**
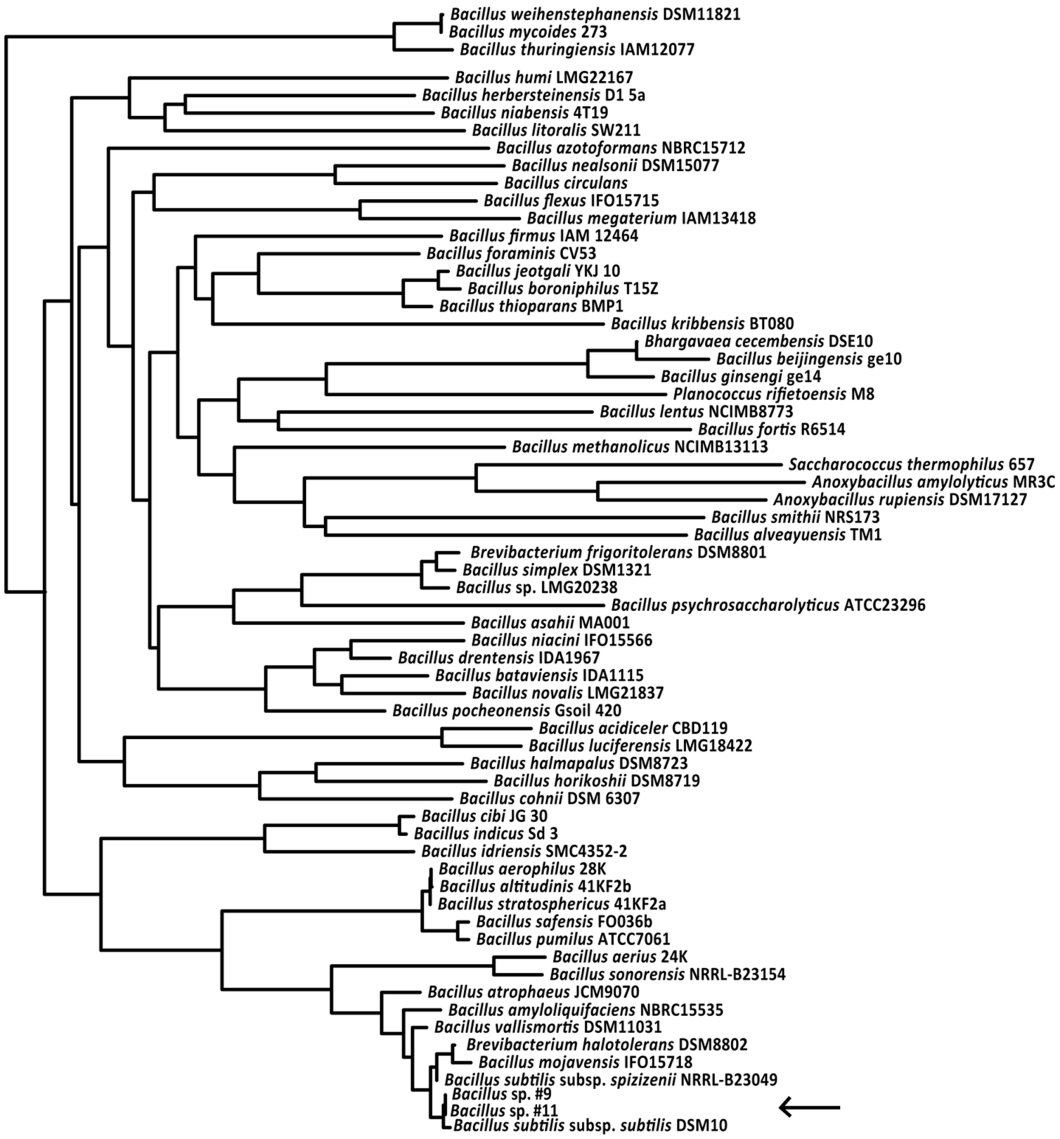
A 16S rRNA gene phylogeny placing *Bacillus* #9 and #11 (indicated with an arrow) in a global *Bacillus* phylogeny, showing that *Bacillus* sp. associated with fungus-growing termites are indistinguishable from *B. subtilis* based on the full-length 16S rRNA gene.

**Figure 4:**
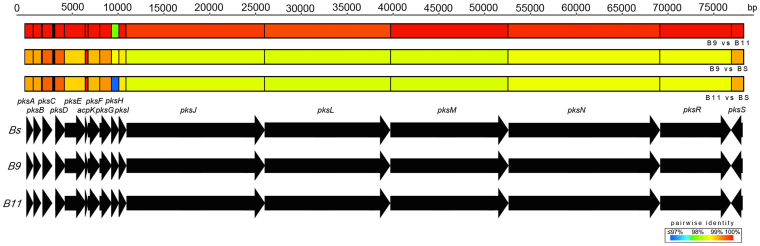
Comparative analyses of the bacillaene gene cluster between *B. subtilis* 168 (Bs), *Bacillus* #9 (B9) and *Bacillus* #11 (B11). Sixteen genes code for the *pks* gene complex involved in bacillaene A (**1**) biosynthesis, and these are *acpK, pksA*, *pksB, pksC, pksD, pksE, pksF, pksG, pksH, pksI, pksJ, pksL, pksM, pksN, pksR* and *pksS*^36^. The gene cluster was identified on a single contig in both #9 and #11. The figure shows a bp-scale bar of the ca. 80 kb gene cluster (top) together with a diagram of the orientation of the genes (bottom) in each of the three genomes. Pairwise comparisons (coloured bars) indicate comparable identities between the termite-associated strains and *B. subtilis*, showing that the two termite strains are more similar (almost identical) to each other across the entire gene cluster.

We obtained whole-genome data for two of the three *Bacillus* strains using mate-paired Illumina HiSeq sequencing, and genomes were assembled using Velvet^32^ and OSLay^33^ and annotated using BASYS^34^. The 16S rRNA genes were extracted from the genomes to obtain a phylogeny placement using the Ribosomal Database Project^35^ (RDP), which identified both strains as being indistinguishable from *Bacillus subtilis* (Fig. 3). Draft genome comparisons revealed that the two strains are almost identical with 4548 shared ORFs being 100% identical at the nucleotide level, 393 shared ORFs being less than 100% identical, but only 17 of these ORFs being less than 97% identical, despite the genomes being only at the draft level (Table 1; Table S1). However, when compared to *B. subtillis* 168, only 402 of the ORFs identified in *Bacillus* #9 are 100% identical to *B. subtillis* and only 455 of the ORFs identified in *Bacillus* #11 are 100% identical to *B. subtillis* (Table S1). We confirmed the presence of the genes necessary for bacillaene production in the two draft *Bacillus* genomes of strains #9 and #11, and compared the 16 *bae* genes coding for the *pks* complex coding for bacillaene A (**1**) to the published sequences obtained for *Bacillus subtilis* strain 168^31^,^36^,^37^,^38^,^39^. The ca. 80 kb gene cluster for the synthesis of bacillaene in both *Bacillus* #9 and #11 also has 16 genes, which are organized identically to those of *B. subtilis* (Fig. 4). Pairwise comparisons of individual *pks* genes between genomes indicated comparable percentage identities between the termite-associated strains and *B. subtilis*. However, the two termite-bacilli strains are more similar to each other across the entire gene cluster (Fig. 4; Table S3). In fact, *Bacillus* #9 and #11 are nearly 100% identical both at the nucleotide and amino acid level in 15 of the 16 genes, with the only exception (98% identical) being the *pksH* gene, which functions to install a methyl group, not part of the backbone of bacillaene (Fig. 4). This implies that termite-*Bacillus* produce the same chemical compound and are more genomically similar to each other, including with regard to the bacillaene gene cluster, than to bacillaene-producing species not associated with fungus-growing termites.

## Discussion

Our findings provide the first evidence of a specific role of bacillaene A (**1**) in a biological system. We obtained this result through optimization of the production and isolation of bacillaene A (**1**), combined with minimizing degradation of the compound by avoiding exposure to light. The compound was initially identified from *Bacillus subtilis* as an antibiotic agent inhibiting prokaryotic protein synthesis^40^. It was reported to display antibacterial activity against various gram-negative (*Escherichia coli*, *Klebsiella pneumoniae*, *Proteus vulgaris*, and *Serratia marcescens*) and gram-positive (*Bacillus thuringiensis* and *Staphylococcus aureus*) bacteria. However, it did not show antifungal activity against the yeasts *Saccharomyces cerevisiae* and *Candida albicans*^40^. Even though the gene cluster and the biosynthesis of bacillaene have been relatively well studied^31^,^41^,^42^, its biological role in nature has been poorly understood, possibly because of its notorious instability^41^. Comprehensive analysis of *Bacillus amyloliquifaciens*, which is a prolific bioactive secondary-metabolite producer, including bacillaene A (**1**), suggested a potential role of *B. amyloliquifaciens* as a defensive symbiont controlling plant pathogens^43^,^44^. Our findings suggest that bacillaene A (**1**) produced by the *Bacillus* obtained from three different colonies of *M. natalensis* could aid in the suppression of antagonistic fungi of the cultivar fungus *Termitomyces*. If so, our findings suggest that i) bacillaene inhibits phylogenetically diverse filamentous fungi, ii) *Bacillus* strains and their secondary metabolites could play a symbiotic role in nature, and iii) *Bacillus* could play a symbiotic role in an ancient mutualism between social insects and fungi.

*Macrotermes natalensis* workers continuously bring in partly degraded plant material (mainly decaying wood) to their colony, and this substrate inevitably harbours microbes that have the potential to compete with or antagonize the termites' mutualistic fungus. The substrate for *Termitomyces* is not directly incorporated into the fungus comb, but experiences obligate gut passage prior to incorporation^17^. The maintenance of defensive gut microbes that can aid in selective inhibition of antagonistic fungi consequently allows for the termites to control the characteristics of the comb substrate to avoid entry of harmful fungi. Our findings suggest that a lineage of *Bacillus* serves a defensive role through the production of a major compound, bacillaene A (**1**) that does not harm the termites' mutualistic fungus, but suppresses the growth of known (*Pseudoxylaria* and *Trichoderma*) and putative antagonistic fungi of the symbiosis. The *Bacillus* lineage has so far been identified in *Odontotermes formosanus*^30^ and *M. natalensis*^this study^, two of the most ecologically important and phylogenetically diverse fungus-growing termite genera^18^. Phylogenetic comparison of the 16S rRNA gene between the *Bacillus* strains identified in this study with those identified by Mathew et al.^30^ (Fig. S6), showed that *M. natalensis* strains were 98.2 ± 0.03% (mean ± SE) similar across the 437 bp fragment to those obtained from *O. formosanus* guts, while they were more distant from isolates from fungus comb (94.1 ± 0.096%). This suggests that the same operational taxonomic unit likely is present in the two termite species and that the *Bacillus* strains we isolated in this study likely originated from the termite gut; however, whether a specific lineage of *Bacillus* associates with the entire Macrotermitinae sub-family remains to be explored.

Bacillaene-producing *Bacillus* has so far been identified in both fungus-growing termite guts and within the fungus comb^30^^, this study^. This suggests that *Bacillus* suppression can take place both during the passage of crude forage through the termite gut, which may allow for partial or complete suppression of incoming fungi, and also later within the fungus comb when *Termitomyces* hyphae decompose the comb substrate. This is possible because *Termitomyces* itself is not adversely affected by bacillaene, which provides an interesting contrast to the utilization of bacteria-derived antifungals in the other major fungus-farming symbiosis: the Neotropical fungus-growing ants (tribe attini). Fungus-growing ants associate with antibiotic-producing Actinobacteria for the suppression of ascomycete *Escovopsis* spp. parasites of the ants' mutualistic fungus^6^,^7^,^8^. The ants maintain the bacteria on specific locations on the ant body^45^, consistent with active use of bacteria-derived compounds during fungus garden hygienic behaviours. In the ants, such tight control of the location and distribution of antifungal compounds may be necessary, because the ants' mutualistic fungus can be inhibited by the bacteria-derived antifungals^46^. In fungus-growing termites, this potential for conflict between fungal and bacterial mutualists of the insect host appears to be avoided, making it possible to maintain the bacterium in both the insect gut and in the fungus comb, potentially allowing for efficient suppression of unwanted microbial contaminants both during preparation and degradation of the fungus garden substrate.

## Methods

### Collections

Three isolates of *Bacillus* (#9, #11 and #13) were obtained from three different *Macrotermes natalensis* colonies collected in Mookgophong (previously Naboomspruit, S24°40′30.5″E28°47′50.4″, elevation 1,045 m), South Africa on the 15^th^ of January 2010. Isolates were obtained by crushing workers in PBS and plating on Chitin (per liter: 4 g chitin, 0.7 g K_2_HPO_4_, 0.3 g KH_2_PO_4_, 0.5 g MgSO_4_·5H_2_O, 0.01 g FeSO_4_·7H_2_O, 0.001 g ZnSO_4_, 0.001 g MnCl_2_ and 20 g agar) or microcrystalline (per liter: 5 g microcrystalline and 20 g of agar) medium. After ca. 14 days of growth on these low-nutrient media, *Bacillus*-like CFUs were transferred to Yeast Malt Extract Medium (per liter: 10 g malt extract, 4 g yeast extract, 4 g glucose, 15 g agar).

### Chemical analyses

*Bacillus* strains #9, #11 and #13 were cultivated in 25 mL YEME liquid medium (per liter: 10 g malt extract, 4 g yeast extract, 4 g glucose) of a 100 mL Erlenmeyer flask with shaking at 200 rpm at 30°C for 2 days. Then 10 mL of culture was inoculated to 1 L of YEME medium in 2.8 L Fernbach flask and cultured at 180 rpm at 30°C for 2 days. 24 L of each (total 72 L for three strains) were prepared and cultured. The liquid cultures were extracted with a total of 72 L of EtOAc. The EtOAc layer was concentrated with a rotary evaporator to yield 3 g of dry extract material. The dry crude extract was re-suspended in MeOH and dried with celite. The celite-adsorbed material was fractionated by column chromatography on C_18_ resin with combinations of MeOH and water (2:8, 4:6, 6:4, 8:2, and 10:0 MeOH to water). Because the 80% fraction was the most active in the antifungal assay, the 80% fraction was further purified through preparative reversed-phase HPLC (Phenomenex Luna column C_18_ (2), 250 × 21.20 mm, UV detection 360 nm, flow rate 10 mL/min). Bacillaene (**1**) eluted at 25 min using isocratic 70% aqueous MeOH with 0.1 formic acid and overall 9 mg of pure bacillaene (**1**) were obtained.

### Antifungal bioassay

For the paper disk diffusion assay on agar plates against *Coriolopsis* (Fungus #8), *Umbelopsis* (Fungus #14), *Fusarium* (Fungus #18), *Trichoderma* (Fungus #22) and *Pseudoxylaria* (Fungus 802-2), 9 cm diameter Petri dishes containing 20 mL of YEME agar medium were used. First, colonies of the fungal strains were inoculated in the center of the YEME agar plate and incubated at 30°C. After 3 days, four 6 mm diameter sterile paper disks were placed on the surface of each petri dish and then imbued with the crude extracts of *bacillus* strains #9, 11 and 13, the fractions of the extracts, and pure bacillaene (**1**) dissolved in DMSO with various concentrations. To set up a negative control, 10 μL DMSO was also added to every petri dish. Every petri dish was monitored daily for 30 days. As bacillaene (**1**) is very unstable under light, the experiment was performed in the dark.

### Genome sequencing and assembly

DNA was extracted from 50 mL LB (tryptone: 10.0 g, yeast extract: 5.0 g, NaCl: 10.0 g) liquid broth culture of each *Bacillus* strain. 1 mL of culture was spun for 20 minutes at 13000 rpm, after which the supernatant was removed. 500 μL of CTAB buffer (10 mL 1 M Tris (pH 8.4), 5 mL 0.5 M EDTA (pH 8), 28 mL 5 M 5NaCl, 2 g cetyltrimethylammonium bromide, 57 mL ddH2O) was then added to each tube. Cells were subjected to two repeated cycles of freezing in −80°C and thawing at 65°C in a heat block. One volume of phenol-choloroform was added to samples, before vortexing and centrifugation for 10 min (13.000 rpm). Supernatants were transferred to clean 1.5 mL eppendorf tubes. 400 μL of cold 100% isopropanol were used for precipitation. After another round of centrifugation (20 min at 13,000 rpm), the pellet of DNA was washed with 70% ethanol and re-suspended in 50 μL ddH_2_O. Whole-genome sequences were achieved using mate-paired Illumina HiSeq at Beijing Genomics Institute (www.genomics.cn) and genomes were assembled using Velvet^32^, checked using Hawkey^47^ (MUMmer 3 package) and following the assembly, contigs were oriented and assembled to supercontigs using the OSLay software^33^ with the *B. subtilis* genome sequence (ATCC 7003, AP012496) as a reference genome. Gene annotations and comparisons were performed using the BASYS software^34^ (for full results, see Table S1). Contigs for draft genomes of *Bacillus* #9 and #11 are deposited in GenBank under the accession numbers APMX00000000 and APMW00000000 (the versions described here are the first, APMX01000000 and APMW01000000).

### Comparative bacillaene gene cluster analyses

We confirmed the presence of the genes necessary for bacillaene production in two *Bacillus* strains. The sixteen genes coding for the bae gene complex involved in bacillaene biosynthesis (*acpK, pksA*, *pksB, pksC, pksD, pksE, pksF, pksG*, *pksH, pksI, pksJ, pksL, pksM, pksN, pksR* and *pksS*^36^) were compared to published *bae* sequences from *B. subtilis* (str. 168 AL009126). Gaps in the cluster sequences were closed using Sanger sequencing. DNA was amplified using a combination of primers provided in Table S2. PCR was performed in a final volume of 20 μL using the VWR ready-to-use mix and 1 μL of each primer (10 μM) at the conditions: 94°C for 30 s, followed by 35 cycles of 94°C for 30 s, 55°C for 30 s and 72°C for 60 s, and final extension at 72°C for 5 min. 5 μL of each PCR product was run on a 1% agarose gel containing 1x GELRED for 30 min. PCR product (15 μL) was purified using the Invitek kit, reeluted in sterilized milliQ water and sent to MWG for sequencing. Comparisons of bacillaene gene similarities between strains at the nucleotide level were made using nucmer^47^ (implemented in MUMmer 3 package) and blastn^48^ (Blast 2.2.27+) (Fig. 4; Table S3). All sequences have been deposited to GenBank (Accession numbers KC832420-KC832451).

## Author Contributions

S.U., A.F., P.S., D.C.O. and M.P. designed the experiments; M.P. collected samples; S.U., A.F. and P.S. performed the experiments; D.C.O. and M.P. performed the general supervision of the project. D.C.O. and M.P. organized and drafted the paper with all authors contributing to the discussion of the data and to the writing.

## Supplementary Material

Supplementary Information Supplementary information

Supplementary Information Table S1

Supplementary Information Table S2

Supplementary Information Table S3

## Figures and Tables

**Table 1:** Genome characteristics of *Bacillus* associated with *Macrotermes natalensis*

	*Bacillus* #9	*Bacillus* #11
Median coverage depth	157.1	155.9
Number of nodes	733	1051
n50/max/total	23077/114783/3958212	11138/62079/3932419
Number of reads used	11407671/11598752	11207443/11250000
Number of supercontigs	22	26
Number of gaps in supercontigs	265	509
Percentage covered	93%	89%
ORFs identified and annotated	4590	4879
Genome draft length (bp)	4101765	4244208
