# Supplementary material for: The fungus-growing termite Macrotermes natalensis harbors bacillaene-producing Bacillus sp. that inhibit potentially antagonistic fungi
Source: Sci Rep. 2013 Nov 19;3:3250. doi: 10.1038/srep03250 (PMC3832938; doi:10.1038/srep03250)
Supplement: Supplementary Information [file srep03250-s1.pdf]

Supplementary information:

The fungus-growing termite *Macrotermes natalensis* harbors bacillaene-producing *Bacillus* sp. that inhibit potentially antagonistic fungi

Soohyun Um[a], Antoine Fraimout[b,c], Panagiotis Sapountzis[b], Dong-Chan Oh[a,1] and Michael Poulsen[b,1]

Fig. S1. Agar plate antifungal assay. 10, 20 and 50μL of 10 mg/mL bacillaene and DMSO control solution on each paper disk testing inhibition of the growth of *Coriolopsis* (a) , *Fusarium* (b), *Pseudoxylaria* (c), *Trichoderma* (d) and *Umbelopsis* (e)

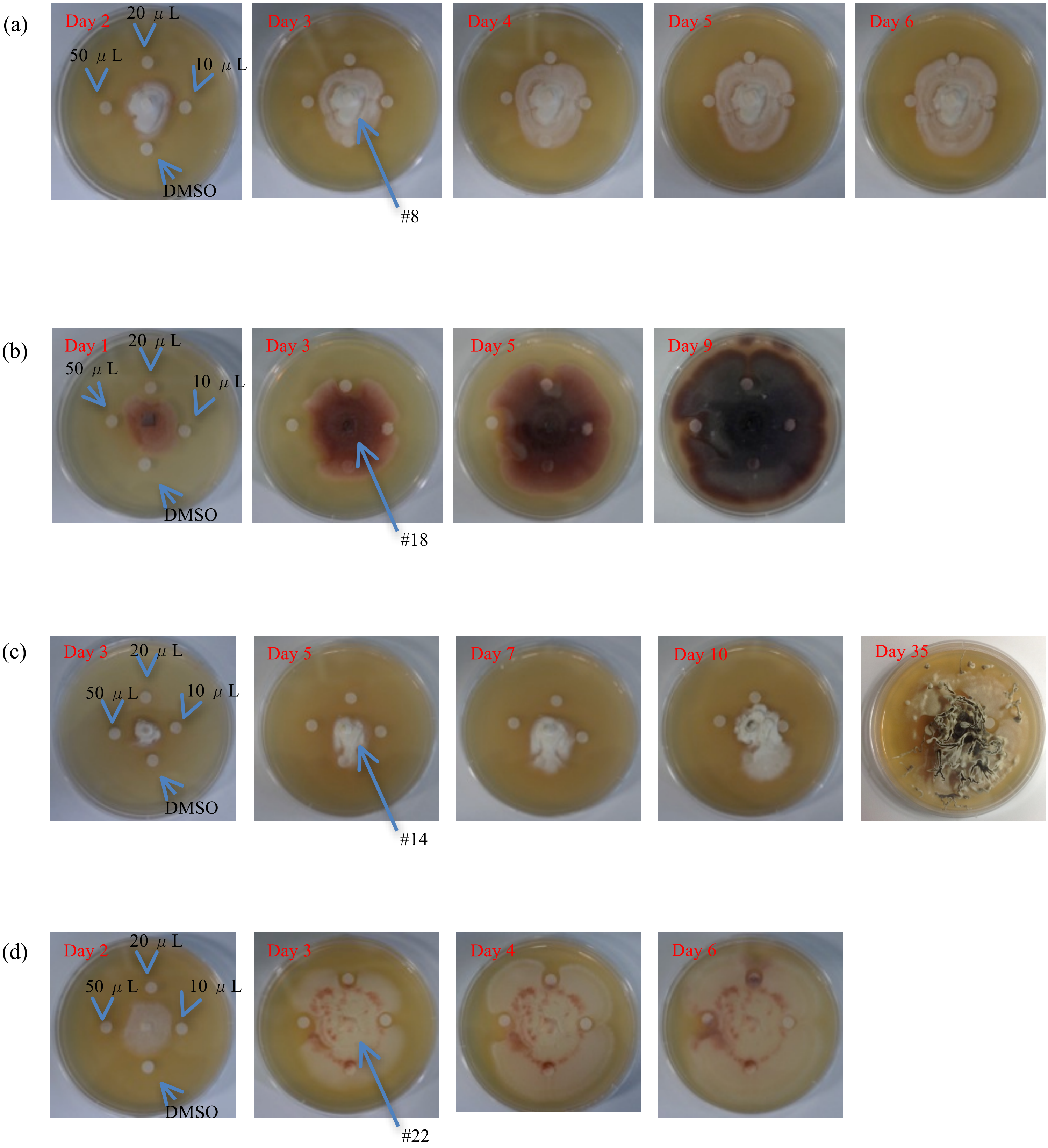

(e)

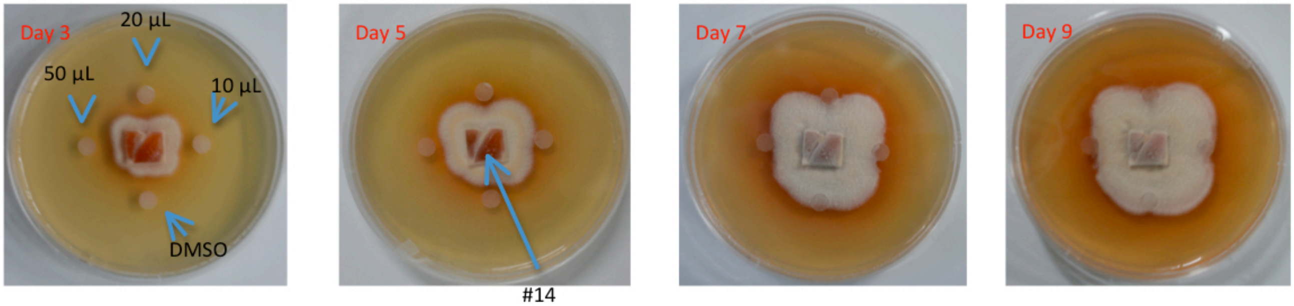

Fig. S2. 1H NMR spectrum of bacillaene (**1**) in CD_3OD

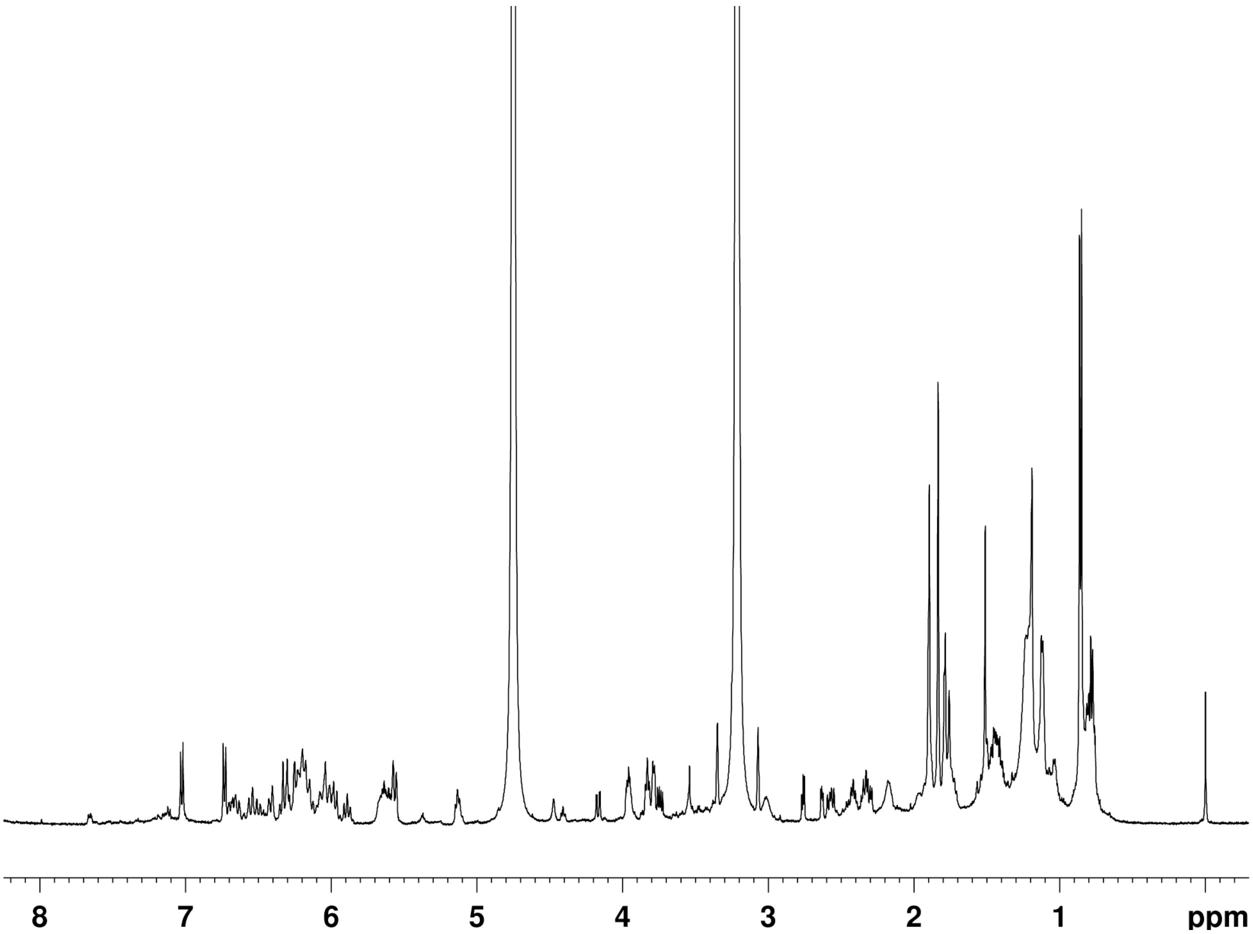

Fig. S3. COSY spectrum of bacillaene (**1**) in CD_3OD

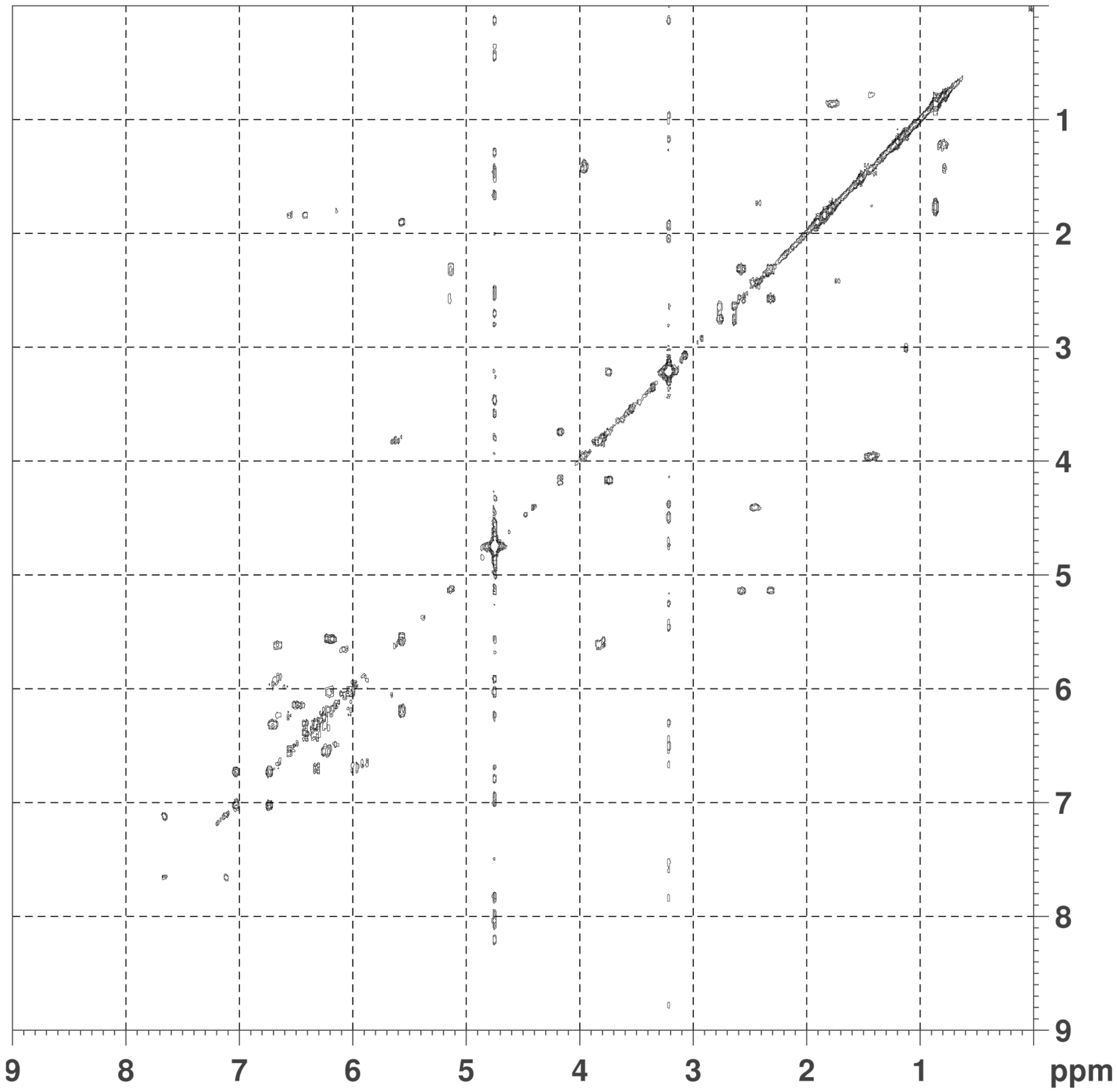

Fig. S4. HSQC spectrum of bacillaene (**1**) in CD_3OD

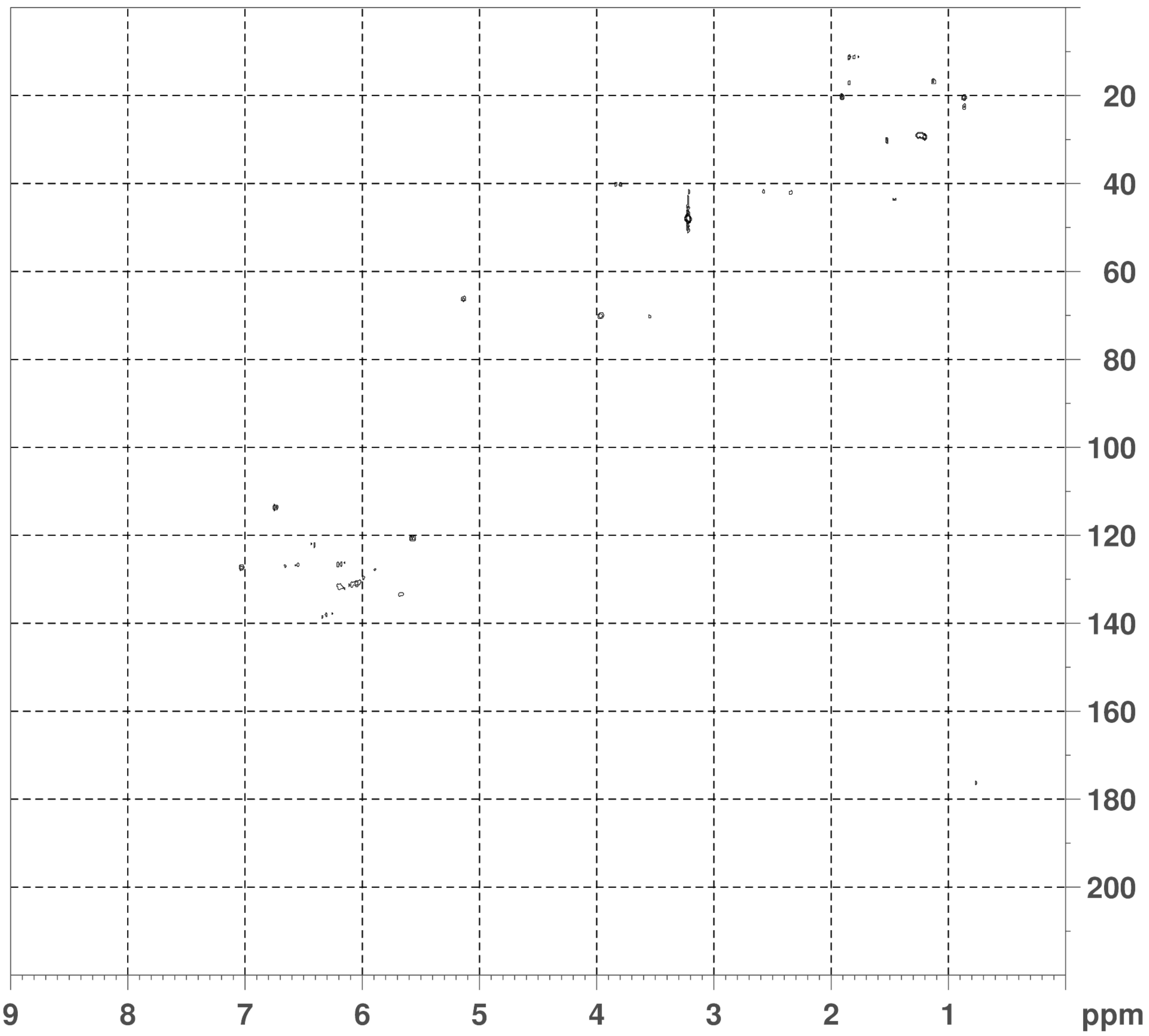

Fig. S5. HMBC spectrum of bacillaene (**1**) in CD_3OD

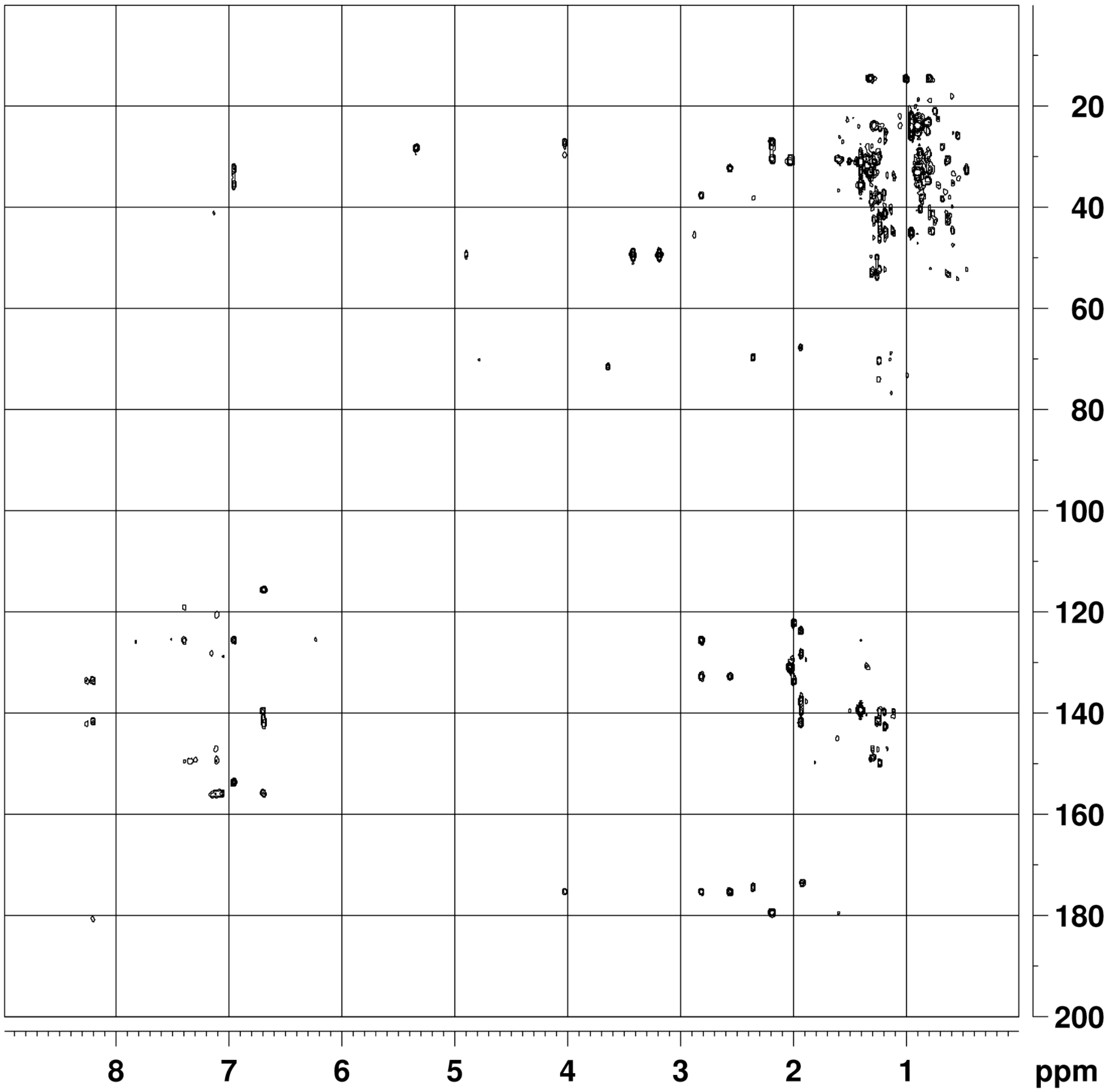

Fig. S6. A neighbour-joining phylogeny with Maximum Likelihood bootstrap support (1000 pseudoreplicates) based on alignment of 437bp overlapping sequences of 16S rRNA from Mathew et al. (2011) (JN000910-15; JN000919-21, JN000923, JN000926-27 and JN000929) showing the placement of *Bacillus*#9 and #11 closest to gut isolates, as well as a matrix of percentage pairwise genetic similarities between sequences.

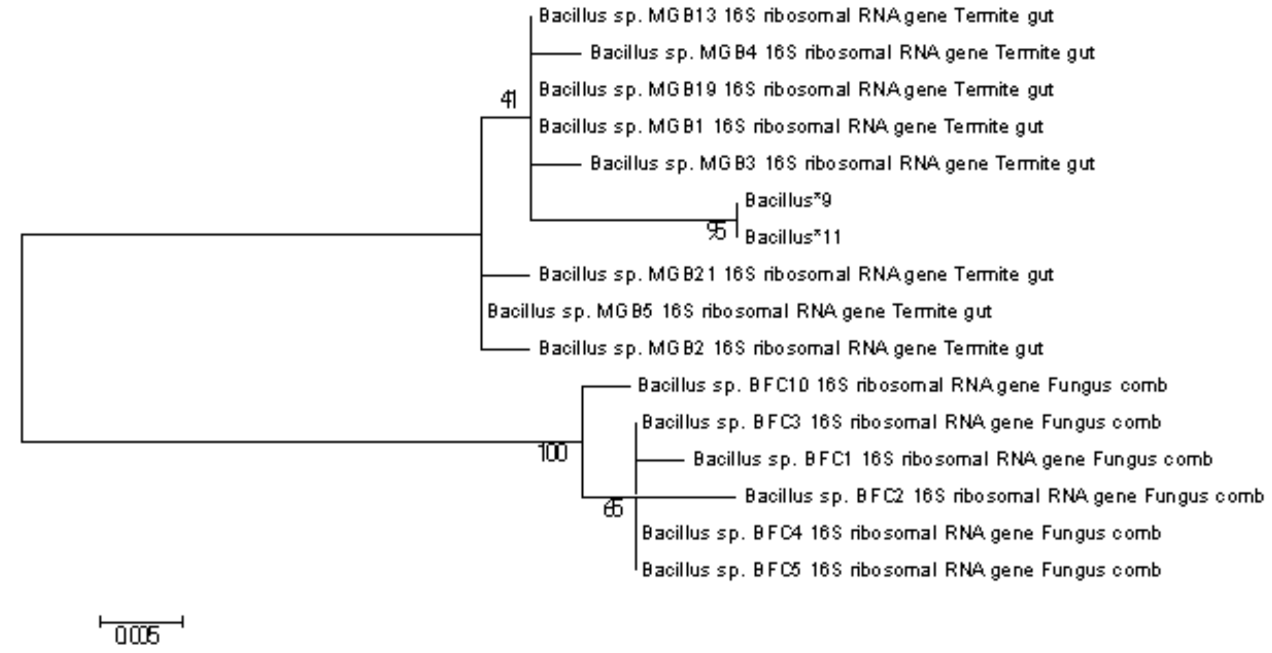

% pairwise genetic distances	Bacillus#9	Bacillus#11	Bacillus sp. MGB3 Termite gut	Bacillus sp. MGB13 Termite gut	Bacillus sp. MGB2 Termite gut	Bacillus sp. MGB19 Termite gut	Bacillus sp. MGB5 Termite gut	Bacillus sp. MGB1 Termite gut	Bacillus sp. MGB4 Termite gut	Bacillus sp. MGB21 Termite gut	Bacillus sp. BFC1 Fungus comb	Bacillus sp. BFC5 Fungus comb	Bacillus sp. BFC2 Fungus comb	Bacillus sp. BFC3 Fungus comb	Bacillus sp. BFC10 Fungus comb	Bacillus sp. BFC4 Fungus comb
Bacillus#9	100.0															
Bacillus#11	100.0	100.0														
Bacillus sp. MGB3 Termite gut	98.1	98.1	100.0													
Bacillus sp. MGB13 Termite gut	98.3	98.3	99.8	100.0												
Bacillus sp. MGB2 Termite gut	98.1	98.1	99.5	99.8	100.0											
Bacillus sp. MGB19 Termite gut	98.3	98.3	99.8	100.0	99.8	100.0										
Bacillus sp. MGB5 Termite gut	98.3	98.3	99.8	100.0	99.8	100.0	100.0									
Bacillus sp. MGB1 Termite gut	98.3	98.3	99.8	100.0	99.8	100.0	100.0	100.0								
Bacillus sp. MGB4 Termite gut	98.1	98.1	99.5	99.8	99.5	99.8	99.8	99.8	100.0							
Bacillus sp. MGB21 Termite gut	98.1	98.1	99.5	99.8	99.5	99.8	99.8	99.8	99.5	100.0						
Bacillus sp. BFC1 Fungus comb	94.0	94.0	95.0	95.0	94.7	95.0	95.0	95.0	94.7	94.7	100.0					
Bacillus sp. BFC5 Fungus comb	94.3	94.3	95.3	95.3	95.0	95.3	95.3	95.3	95.0	95.0	99.5	100.0				
Bacillus sp. BFC2 Fungus comb	94.0	94.0	95.0	95.0	94.7	95.0	95.0	95.0	94.7	94.7	99.0	99.5	100.0			
Bacillus sp. BFC3 Fungus comb	94.5	94.5	95.4	95.4	95.2	95.4	95.4	95.4	95.2	95.2	99.5	100.0	99.5	100.0		
Bacillus sp. BFC10 Fungus comb	93.5	93.5	93.8	93.8	93.5	93.8	93.8	93.8	93.5	93.5	99.4	99.7	99.1	99.7	100.0	
Bacillus sp. BFC4 Fungus comb	94.3	94.3	95.3	95.3	95.0	95.3	95.3	95.3	95.0	95.0	99.5	100.0	99.5	100.0	99.7	100.0

Table S1: BASYS gene-by-gene similarities between *Bacillus* #9, #11 and *B. subtilis* strain 168 (separate excel file: Um_TableS1).

Table S2: Primers used to close gaps in the bacillaene gene cluster (separate excel file: Um_TableS2).

Table S3: Pairwise Blastn and Blastp percentage similarity of the 15 *bae* genes in the bacillaene gene cluster between *Bacillus* #9 and #11 and *B. subtilis* strain 168 (separate excel file: Um_TableS3).
